# On Anæsthetics

**Published:** 1866-08

**Authors:** Erastus Wilson

**Affiliations:** 44 Bond Street, New York


### ON ANÆSTHETICS.

Permit me, through the columns of the REPORTER, to contribute a few remarks upon the subject now attracting some attention, of the comparative merits of nitrous-oxide and other anæsthetics.

Almost since the first general introduction of anæsthetics into surgical application, (fifteen or sixteen years,) I have been in almost daily experience with them, that experience embracing most of the substances employed to produce anæsthesia, either general or local.

On the recent re-introduction of nitrous-oxide for that purpose, I made careful experiments with it, both in its preparation and its application, my experience rising in the aggregate to some hundreds of successful cases, (teeth extraction,) yet I am not prepared to give it so sweeping a preference as does Dr- CARNOCHAN over chloroform and ether. I am not of those who resent all innovation of established practice, but while we lean generously toward the *new*, let us at least not be unjust to the *old*.

I am aware that novelty has an almost irresistible charm even to the most matured judgements, and that the well-known and very laudable desire of our representative men for progress, may sometimes lead them into undue enthusiasm in prematurely signalizing an advance, is not altogether unnatural; but while we are proud of their enterprise, let us beg of them that they will not commit themselves too fully upon very limited data. It seems to me that the data in regard to nitrous-oxide as an anæsthetic, are far too limited to admit, in fairness, of any conclusive comparison of its merits with those of chloroform and ether.

Furthermore, I wish to remark that we are accustomed to speak of the danger to life connected with the inhalation of ether and chloroform as being inherent in these agents, and I wish to inquire of your readers if, in the general private opinion of the profession, this is really the case.

There have been deaths occuring under their administration —no one doubts that; but, *entre nous*, have none occurred from the bungling or unskilful use of the surgeon's knife?

Who is prepared to say that a larger proportion can be justly placed to the charge of the one than to that of the other?

Yet I apprehend that no one would aver that the danger to life was or is inherent in the knife, instead of the hand that guides it, and that its use should be abolished in consequence; at least, until the proof should be clearly established that we have more than its equivalent with which to replace it.

We should rather seek increased skilfulness in its employment, and in my humble opinion, we are bound to render to ether and chloroform the same justice, instead of making them the scape-goats of our own shortcomings. I, for one, am not satisfied that the best skill we are capable of attaining is generally employed to guard against the dangers that might be intelligently foreseen in the administration of these agents, powerful for good or for evil—ether and chloroform.

My own experience leads me to be most guarded against the approach of danger in the three following directions, viz.

1st. *Asphyxia*—from privation of atmospheric air.

2d. *Hyper-Anæsthesia.*

3d. *Nervous shock*—from too forced and sudden administration of anæsthetic influence; or from mental terror, inspired by visible preparations, excitement of sympathizing friends, and forcing the patient to submit without first quieting those fears.

These, I am convinced, are the real sources of danger in the employment of chloroform and ether, and they seem to my mind, if not *absolutely*, at least to a very great extent *avoidable.*

If I were asked how, I would reply that in the avoidance of the first named of these dangers, which is by no means imaginary, the precaution is very simple and obvious, consisting of the admittance of sufficient atmospheric air to support respiration.

In this connection, I must conscientiously apply the strongest terms of condemnation to the custom, so largely in vogue, of smothering down the subject, as some term it, for here we incur the two principal dangers combined in one. I know of nothing more calculated than such a struggle for respiration, to throw the subject into a panic of mental terror, such as a delicate organization would not be able to survive.

Against the danger arising from too sudden and from over-etherization, the precautions are, in my view, nearly identical, and first of all, should be to secure the confidence of the patient.

In my own hands, in proportion to the calmness and confidence with which the patient has approached the operation, has been *cœteris paribus*, the absence or otherwise of unfavorable symptoms.

If it is approached with terror, the patient yields tardily, is not easily subdued, the symptoms of hyper-anæsthesia will often immediately follow, requiring the closest attention to prevent the strong tendency to paralysis of the vital functions.

I employ by preference chloroform, formerly DUNCAN & FLOCKHAST'S; at the present time, I find fully its equivalent in that prepared by EDWARD R. SQUIBB, of Brooklyn, L. I.

Having taken the usual precautions in regard to the conditions of the stomach and dress, so as to have no obstructions of the vital machinery, with my finger upon the pulse, and with calm words of assurance, and closely watching the vital movements, I place the anæsthetic just near enough to perceive its effect upon the system, graduating this so as to introduce it very slowly and evenly until the voluntary muscles are relaxed, and the subject in a quiet and perfect anæsthesia.

In this manner, I have no excitement, no struggle, no spasm, no asphyxia, no hyper-anæsthesia, and very little nervous derangement, either present or following.

I in fact find less nervous disturbance with chloroform than with ether, and under the precautions I have indicated, I know of no reason to think it less *safe;* if any one does know of such reason, I would gladly be informed of it. I could cite many cases to prove the important rôle which *fear* and *confidence* enact in this connection, but I presume it to be patent to every intelligent observer, and I desire that my letter shall be acceptably brief. ERASTUS WILSON, M. D.

Of 44 Bond street, New York.

*Havana, Cuba, April 19th,* 1866.

---

Remarkable results have been obtained by M. SCHLŒSING in the production of exceedingly high temperature by the combustion of gas with air. By regulating the quantity of hydrogen and air brought together at the time of combustion, a considerable range of temperature can be obtained, the highest named in a communication recently made to the Academie des Sciences of Paris, by ST. CLAIRE DEVILLE, being 2736 deg. Cent.

